# Meta-Analyses of QTLs Associated with Protein and Oil Contents and Compositions in Soybean [*Glycine max* (L.) Merr.] Seed

**DOI:** 10.3390/ijms18061180

**Published:** 2017-06-01

**Authors:** Kyujung Van, Leah K. McHale

**Affiliations:** 1 Department of Horticulture and Crop Science, The Ohio State University, Columbus, OH 43210, USA; van.71@osu.edu; 2 Center for Soybean Research, The Ohio State University, Columbus, OH 43210, USA; 3 Center for Applied Plant Sciences, The Ohio State University, Columbus, OH 43210, USA

**Keywords:** amino acids, fatty acids, meta-analysis, oil, protein, quantitative trait loci, soybean

## Abstract

Soybean [*Glycine max* (L.) Merr.] is a valuable and nutritious crop in part due to the high protein meal and vegetable oil produced from its seed. Soybean producers desire cultivars with both elevated seed protein and oil concentrations as well as specific amino acid and fatty acid profiles. Numerous studies have identified quantitative trait loci (QTLs) associated with seed composition traits, but validation of these QTLs has rarely been carried out. In this study, we have collected information, including genetic location and additive effects, on each QTL for seed contents of protein and oil, as well as amino acid and fatty acid compositions from over 80 studies. Using BioMercator V. 4.2, a meta-QTL analysis was performed with genetic information comprised of 175 QTLs for protein, 205 QTLs for oil, 156 QTLs for amino acids, and 113 QTLs for fatty acids. A total of 55 meta-QTL for seed composition were detected on 6 out of 20 chromosomes. Meta-QTL possessed narrower confidence intervals than the original QTL and candidate genes were identified within each meta-QTL. These candidate genes elucidate potential natural genetic variation in genes contributing to protein and oil biosynthesis and accumulation, providing meaningful information to further soybean breeding programs.

## 1. Introduction

Although significant crop improvements have been made internationally, there is still demand for increased food supply and quality [1]. In addition to traditional phenotypic selection, molecular breeding, often facilitated by genome sequence analysis, has been a primary tool for development of new cultivars to increase food security [1]. Like many other legumes, the demand for soybean has increased due to its nutritional importance in animal feed, potential use as an industrial raw material, for its benefits to human health [2], and use as a fuel feedstock [3].

Because soybean seed contains approximately 40% protein and 71% of the world’s meal consumption is reliant on soybean, a percentage which is increasing [4], total protein content has been one of the primary quality traits on which soybean breeding has focused. Both human and animal consumption of soybean is predicted to increase as the demand for plant and animal protein as well as the world population grows [5]. About 77% of animal meal consumption is dependent on soybean meal, used as a source of protein and specific amino acids. Thus, soybean breeding programs have initiated development of new soybean cultivars which possess essential and balanced amino acid compositions, such as increased levels of cysteine (Cys), methionine (Met), lysine (Lys) and threonine (Thr) [5].

With 61% of the world’s oilseed production dependent on soybean [4], oil accumulation in soybean seeds has also been a critical focal point of breeding efforts for soybean grain quality. Efforts towards the genetic analyses of oil content and composition in soybean seed have been increasing due to the high demand of vegetable oils and its role in human health [6]. Due to the relatively short shelf life of soybean oil, the development of cultivars with modified oils possessing decreased linolenic acid and elevated oleic acid has become a critical breeding goal [7]. Additional modified oil targets have also been proposed, potentially resulting in high-value soybean oils [8,9]. Thus, knowledge of the genetic control of total protein and oil content as well as specific compositions can be important for the development of prescriptive soybean cultivars.

Over the past 25 years, mapping studies utilizing biparental populations have been carried out for the identification of quantitative trait loci (QTL) for soybean seed composition. Through these studies, hundreds of loci contributing to the protein and oil contents and compositions of seed have been identified, as well as few molecular markers which have been successfully applied in marker-assisted breeding [5,10,11,12,13]. Of the more than 700 QTLs identified from over 60 studies mapping protein and oil seed contents, only 57 QTLs have been confirmed (available online: http://soybase.org). To gain the official designation of a confirmed QTL, the QTL must be evaluated in an independent set of meiotic events sharing at least one parent with the original population, in independent environments, and at an experiment-wise error rate of less than 0.01 (available online: http://soybase.org). While the criteria to confirm a QTL are not always met, there are frequently overlapping QTLs that have been identified from different genetic sources and or at an experiment-wise error rate of greater than 0.01. However, it is not always simple to identify common QTL regions for specific traits due to various factors, including the marker sets, statistical methods, parents, size and generation of populations, experimental design, and environment which can each influence the detection, location, and level of significance of QTLs. Thus, meta-analyses, which utilizes a maximum likelihood estimation with consideration of population size and QTL positions and effects, have been applied to consolidate and refine locations of robust QTLs identified for the same trait [14].

Meta-analysis has been used for studying QTLs for various traits in many major crops; drought tolerance in rice [15], yield-related traits in wheat [16], agronomic traits in cotton [17], flowering time in maize [18] and abiotic stress tolerance in barley [19]. Several meta-QTL analyses have been carried out in soybean and have included traits such as resistance to soybean cyst nematode [20], plant height [21], 100-seed weight [22] and delayed-canopy-wilting [23]. Contents of soybean seed protein [24] and oil [25] have also been previously studied by meta-analysis with 107 and 121 QTLs, respectively. However, in recent years numerous additional QTL mapping studies for these two traits have rapidly accumulated [10,11,12,13,26,27,28,29,30,31,32,33,34,35,36], as well as, QTL studies on the amino acid and fatty acid compositions of soybean seed protein and oil [37,38,39,40,41,42,43]. Thus, with a nearly three-fold increase in the number of QTL available, a new meta-analysis for seed protein and oil contents which includes recent QTLs for seed protein and oil content as well as seed amino acid and fatty acids profiles is timely.

In this study, we collected the information related to the genetic location and effects of QTLs for seed contents of protein and oil and the respective profiles of amino acids and fatty acids from over 80 studies. Using BioMercator V. 4.2 (Versailles, France), meta-QTL analyses were performed with the genetic information from over 600 QTLs (175 QTLs for protein, 205 QTLs for oil, 156 QTLs for amino acids and 113 QTLs for fatty acids), allowing for identification of meta-QTLs, which represent robust QTLs identified in multiple studies and with narrowed confidence intervals (CI) relative to the original QTLs. Candidate genes were identified for each of the meta-QTLs. Findings from these meta-QTL analyses along with identified candidate genes provide insight into soybean’s naturally varying genes involved in the biosynthesis and accumulation of protein and oil in the seed and will be useful for marker-assisted soybean breeding.

## 2. Results

### 2.1. Collection of QTLs for Soybean Seed Protein and Oil Contents and Compositions

SoyBase (available online: http://soybase.org) and recent literature were mined up to 2016, inclusive, to identify all map and QTL information for soybean seed protein and oil contents and amino acid and fatty acid profiles. A total of 84 studies were identified (Table S1) which reported a total of 1320 QTLs for protein, oil, amino acids and fatty acids, 648 QTLs with no R2 value and no genetic map information were excluded (Table S2). Thus, a total of 672 QTLs (184 for protein, 212 for oil, 156 for amino acids and 120 for fatty acids) were used in our meta-analyses (Table S2). Of these QTLs, 23 had LOD scores below 2.0, as suggested by Qi et al. [24] these were removed and separate meta-QTL analyses were conducted with only “high confidence” QTLs, which consisted of 649 QTLs (175 QTLs for protein, 205 QTLs for oil, 156 QTLs for amino acids and 113 QTLs for fatty acids) with LOD scores > 2.0 (Table S2).

### 2.2. QTL Projection on a Soybean Consensus Map and Meta-Analysis of Seed Compositions QTL

The soybean consensus map 4.0 [44] was used as a reference map for the projection of QTLs. Of the 672 QTLs (649 high confidence QTLs), 284 QTLs (263 high confidence QTLs) were able to be projected on the reference map. Though not evenly distributed, projected QTLs were positioned on all 20 chromosomes. With 38 (35 high confidence) projected QTLs, chromosome (Chr) 20 had the largest number of projected QTLs, whereas only three QTLs were projected on Chr 11 (Table S3). Overall, more than half of the QTLs were not projected on the reference map. Failure to project a QTL is caused by a lack of common marker pairs which flank the QTL in the original and soybean reference maps. For example, meta-analysis of amino acid QTLs was initiated with 156 QTLs which were primarily mapped with SNP markers which were largely unique to these studies, thus, only 9 QTLs were able to be projected (Chrs 1, 6, 9 and 20; Table S3).

Using BioMercator v4.2, meta-analyses were performed for seed protein and oil content separately. Meta-analyses of amino acid composition and fatty acid composition were carried out in two groups, respectively. Soybean seed protein and oil contents have a well-established negative correlation [10,45,46] and seed protein versus Cys concentration and seed protein versus Met concentration have also been reported as displaying positive or negative correlations depending on the study [5,11,38,42,47,48]. Therefore, meta-analyses of QTL for these traits known to co-vary were also carried out in predetermined combinations of protein and oil (protein+oil) and protein, Cys and Met (protein+Cys+Met).

In the first step of meta-analysis (Meta-Analysis step 1 of 2) [49], for each chromosome and trait, the best meta-QTL models with K consensus QTLs was selected according to the minimum Akaike Information Criterion (AIC) (Tables S3 and S4). With the model chosen, meta-QTLs were successfully generated from six chromosomes with the most likely positions and CIs calculated in the second step of meta-analysis (Meta-Analysis step 2 of 2) (Tables S3 and S4) [49].

**Figure 1:**
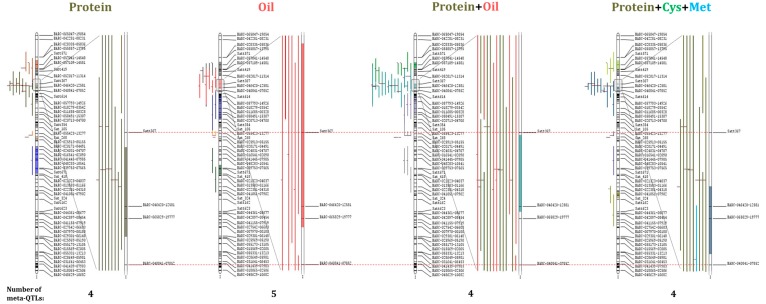
Comparisons among protein, oil, protein+oil and protein+Cys+Met meta-quantitative trait loci (meta-QTLs) on Chromosome (Chr) 20 (Linkage group I; LG I). Projected QTL are displayed to the left of Chr 20 for each trait or trait combination. A 95% of confidence interval of each meta-QTL is represented as filled colors on the chromosome arm, with four, five, four and four meta-QTLs identified for protein, oil, protein+oil and protein+Cys+Met, respectively. A detailed view of the projected QTL contributing to each meta-QTL cluster #2 of Chr 20 (Table 1) are also shown on the right of the chromosome. In the detailed view, each bar represents a projected QTL and is color-coded for each trait (protein, khaki; oil, red; cysteine, green; and methionine, blue).

**Figure 2:**
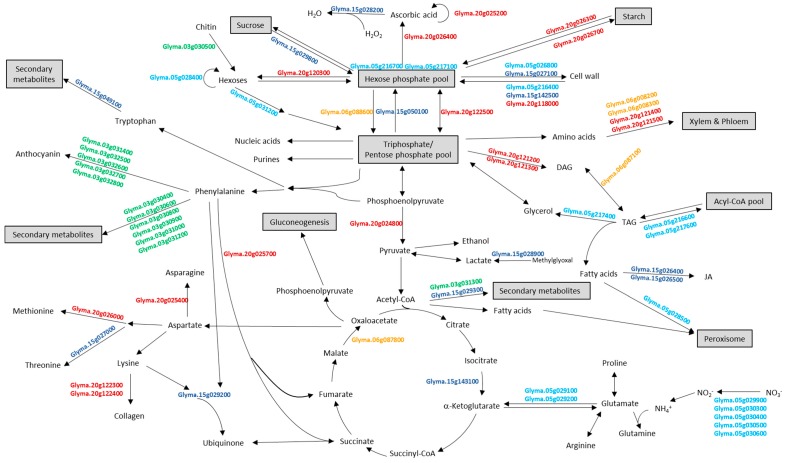
Overview of metabolic processes involved by positional and functional candidate genes. Candidate genes names from Glyma.Wm82.a2.v1 (Gmax2.0) (available online: http://soybase.org) are shown in bold and color-coded by chromosomes.

A total of 55 meta-QTLs (43 high confidence meta-QTLs) were identified for all traits and trait combinations (Table 1). The meta-QTLs represented 284 projected QTLs (263 high confidence projected QTLs). While the majority of meta-QTLs were identified only when the meta-analysis was carried out across traits and included both protein and oil QTLs (Figure 1 and Table S4), Chr 20 possessed high confidence meta-QTLs for protein, oil, protein+oil and protein+Cys+Met (designated as mP20-#, mPO20-#, and mPCM20-#, respectively) (Figure 2 and Table S4). Twelve meta-QTLs on Chrs 9, 14, and 15 were identified only when QTLs with LOD scores < 2.0 were included in the analysis (Table 1). Meta-analysis reduced the CI of meta-QTLs relative to the projected QTLs by a wide margin, with the projected QTLs spanning an average of 21.77 cM (30.07 cM for high confidence QTLs) and meta-QTLs spanning an average of only 3.88 cM (4.30 cM for high confidence meta-QTLs).

### 2.3. Identification of Candidate Genes for Each Meta-QTL

In order to identify positional candidate genes potentially contributing to the meta-QTLs for each trait, the physical and genetic positions of the left and right markers of each meta-QTL’s CI were obtained for those markers found on both the consensus map 4.0 [44] and *G. max* genome assembly, version Glyma.Wm82.a2.v1 (Gmax2.0) (available online: http://soybase.org). For those markers not positioned on either the soybean consensus map 4.0 or Gmax2.0, the next nearest marker located on both the consensus map and Gmax2.0 was selected. The meta-QTLs encompassed a total of 7412 positional candidate genes (5440 positional candidate genes for high confidence meta-QTLs), with each meta-QTL encompassing an average of 135 positional candidate genes (127 positional candidate genes for high confidence meta-QTLs) (Table 1). Meta-QTL mPO20-2 contained the smallest number of positional candidate genes, with 14 genes within the CI of 1.83 cM (24.07–25.90 cM on Chr 20, Figure S2), whereas there were an immense 590 positional candidate genes encompassed by mPO7-6 with a CI of 17.41 cM (57.99–75.40 cM on Chr 7). While, generally, a small number of positional candidate genes were detected if the CI of the meta-QTL was small, the CIs of mP20-3, mO20-4, mPO20-4 and mPCM20-3 were only 0.61, 0.01, 0.00 and 0.14 cM, respectively; however, 49 positional candidate genes were identified within each of these meta-QTL. This was largely due to the closest marker being located at least 4 cM away on the reference map from the CI boundaries (Table 1). Thus, while the CIs of meta-QTL are generally narrower than the projected QTL from which they are synthesized, the advantage of this narrowed region in identifying positional candidate genes can only be observed when the markers can be easily translated to the genome sequences (Gmax2.0).

For the 14 meta-QTLs having less than 50 positional candidate genes, detailed functional information of candidate genes from each meta-QTL were obtained from Phytozome v. 12.0 (available online: http://www.phytozome.net; Table S5). Interestingly, each meta-QTL encompassed at least one positional candidate gene annotated with potential metabolic functions relating to protein and oil biosynthesis and/or accumulation, such as sucrose biosynthesis, glycolysis, gluconeogenesis, amino acids biosynthesis/degradation, etc. (Table 2 and Figure 2).

## 3. Discussion

### 3.1. Meta-Analysis Aids in the Identification of Robust QTLs and Narrowing of Confidence Intervals

Meta-analysis of QTLs was developed by Goffinet and Gerber [14] in order to assist in identification of consistent and robust QTLs and to improve the precision of their genetic locations. The composition of soybean seed is a well-studied trait with numerous genetic studies having been carried out to identify QTLs for protein and oil contents and amino acid and fatty acid compositions (Table S1). A meta-analysis can aid in the synthesis of these myriad QTLs in order to identify the genetic regions robustly associated with each trait in multiple environments and genetic backgrounds. Previously, two studies have carried out meta-analyses related to QTLs for soybean seed protein and oil contents. The previous meta-analysis of protein content QTLs identified 23 meta-QTLs on 13 chromosomes with CIs of meta-QTLs ranging from 1.52 to 14.31 cM [24]. In the previous meta-analysis of oil content, 20 meta-QTLs were detected on 13 chromosomes and their CIs ranged from 1.3 to 12.35 cM [25]. The meta-analyses carried out in this study integrate the locations of hundreds of QTLs for seed protein and oil contents and amino acid and fatty acid compositions using maximum likelihood estimation with consideration of population size and additional QTL information. We have projected 284 of 672 QTLs for these traits onto a single reference map and integrated these into 55 meta-QTLs for protein, oil, protein+oil, and protein+Cys+Met to provide a genetic framework for seed protein and oil contents (Table 1).

No meta-QTLs were synthesized for fatty acids or amino acids when analyzed as independent traits. This may be due to the limited number of studies as compared to protein and oil as well as the minimal overlap of QTLs detected among those studies. However, genes which contribute to environmentally stable changes in fatty acid composition in multiple genetic backgrounds have been cloned, such as 3-keto-acyl-ACP synthase II gene (*KAS II*) [50], ω-3 fatty-acid desaturase gene (*FAD3*) [51], microsomal oleate desaturase gene (*FAD2*) [52] and Δ^9^-stearoyl-ACP-desaturase gene (*SACPD-C*) gene [53]. Thus, it is known that at least a portion of the QTL for fatty acid composition are robust. 

In Chr 20, meta-QTLs consistently possessed narrower CIs than the projected QTLs from which they were synthesized. Meta-QTLs for protein, oil, protein+oil, and protein+Cys+Met decreased CI by 13.56 (73.3%), 21.69 (81.1%), 28.63 (89.6%) and 18.67 (88.7%) cM, respectively, as compared to the projected QTL. The translation of genetic distances to the identification of positional candidate genes on a physical map is dependent on the ability to locate the physical positions of flanking genetic markers from the reference map. Accordingly, we found that the decreased confidence intervals often, but not always (e.g., mP20-3, mO20-4, mPO20-4 and mPCM20-3), correlated with a decrease in the number of positional candidate genes encompassed by the meta-QTLs in comparison to the projected QTLs. Meta-QTLs for protein, oil, protein+oil, and protein+Cys+Met decreased the average number of positional candidate genes by 367 (480 to 113), 686 (815 to 129), 526 (702 to 176) and 461 (527 to 66), respectively, as compared to the projected QTLs.

Traditionally, QTL positions are refined through the tedious process of fine-mapping which can include selecting for recombination in targeted regions and evaluating those individuals (e.g., [35]), increasing marker density (e.g., [54]), and/or “mendelizing” the QTL by generating near-isogenic lines [35,55]. In some cases, meta-analysis of QTLs may replace or strengthen these methods. For example, mPO15-2, formed from eight projected QTL is coincident with the confirmed seed protein QTL cqSeed protein-001 (available online: http://soybase.org) [56]. Satt384 (at 19.62 cM on the soybean consensus map) is positioned within the mPO15-2 CI. Satt384 was the key marker in the fine-mapping of cqSeed protein-001 and fine-mapping had previously served to decrease the interval to 535 kb based on Gmax2.0 [35]. Though the positioning of the mPO15-2 flanking markers on the reference genome are inverted relative to the reference map, making a direct interpretation of results difficult, mPO15-2 coincided with the 535 kb interval identified by fine-mapping and even further narrowed this region (Table 1).

### 3.2. Meta-QTLs Can Be Further Defined and Refined Through the Combined Analysis of Correlated Traits

We found that combining QTLs identified from correlated traits in a meta-analysis resulted in the identification of additional meta-QTLs and further narrowed CIs. Most of the meta-QTLs identified in this study were for combined protein and oil contents. However, on Chr 20, meta-QTLs were detected when projected QTLs from protein and oil traits were analyzed separately, in combination, as well as for combined projected QTLs from the correlated traits protein, Cys and Met (Figure 1 and Table 1). Many studies have demonstrated Chr 20 (LG I) has QTLs with a strong effect on protein content and a lesser effect on oil content in soybean seed [46,57,58]. In our study, six meta-QTL regions were identified on Chr 20. Three meta-QTL regions were identified in all four meta-analyses, protein, oil, protein+oil and protein+Cys+Met. These meta-QTL spanned from approximately 13 to 18 cM (mP20-1, mO20-1, mPO20-1 and mPCM20-1), 22 to 27 cM (mP20-2, mO20-2, mPO20-2 and mPCM20-2), and 49 to 50 cM (mP20-3, mO20-4, mPO20-4 and mPCM20-3). The remaining two meta-QTL regions were only found in some subset of the four meta-analyses (approximately 29 to 41 cM for mO20-3 and mPO20-3, approximately 54 to 67 cM for mP20-4 and mO20-5, and 75.05 to 78.48 cM for mPCM20-4). Thus, there were meta-QTL regions that were identified both only for QTL from a single trait and only for QTL from combined, correlated traits.

By combining the negatively correlated traits of seed protein and oil contents, CIs for Chr 20 meta-QTLs were reduced by an average of 3.32 cM in comparison to meta-analysis of each trait separately (Figure 1 and Figure S2). This narrowing of CIs resulted in a decrease in the number of positional candidate genes encompassed by each meta-QTL. This is exemplified by mP20-2, mO20-2, and mPO20-2 where the number of positional candidate genes was reduced from 26 and 63, to only 14, respectively (Table 1). Thus, our study suggested that meta-analysis of QTLs from not only single traits but also of correlated traits can be used to identify meta-QTLs with potential pleiotropic effects and result in narrowed CIs.3.3 The incorporation of QTLs with low LODs score into meta-analysis does not hinder analysis and provides confirmation.

Qi et al. [24] suggested the removal of reported QTLs with LOD scores < 2.0 in order to avoid false positives in meta-analysis. However, a number of QTL mapping studies have routinely reported QTLs with lower LOD scores, as in, for example, the application of a chromosome-wide threshold in order to identify “suggestive” QTLs [59,60]. We posited that a meta-analysis of QTLs would provide the opportunity to validate these “suggestive” QTLs. The inclusion of 23 QTLs with LOD scores below 2.0 resulted in the detection of an additional 12 meta-QTLs: four, three and five clusters on Chrs 9, 14 and 15, respectively (Table 1 and Figure S1). Meta-QTLs mPO9-1~4, mPO14-1~3 and mPO15-1~5 were synthesized from 17, 16 and 21 projected QTLs, respectively. As the projected QTL contributing to these meta-QTL were from each from multiple studies [10,11,13,26,27,29,30,31,33,34,57,61,62,63,64,65,66,67,68,69,70,71,72,73,74,75], these QTL with LOD scores below 2.0 have been identified in multiple environments and genetic backgrounds, indicating that they are, indeed, reliable QTL. Thus, meta-analysis can be applied to identify robust QTL from “suggestive” QTL.

### 3.3. Putative Functional Candidate Genes Were Identified from the Positional Candidates Encompassed by Meta-QTLs

Of the 55 meta-QTLs identified, 14 encompassed a limited number (<50) of positional candidate genes. For these 14 meta-QTLs, representing nine distinct regions on Chrs 3, 5, 6, 15 and 20, the positional candidate gene lists were mined for functional candidates based on their gene annotation (Table 2). While functional candidates may include, for example, transcription factors and unannotated genes, here we have focused on metabolic activities that may influence seed protein and oil biosynthesis and accumulation. This lead to the identification of 69 functional candidate genes for these nine meta-QTL regions.

Meta-QTL mPO3-1 possessed 35 positional candidate genes, among which included putative phenylacetaldoxime monooxygenases and anthocyanidin 3-*O*-glucosyltransferases involved in anthocyanin biosynthesis [76] (Table 2 and Figure 2). Phenylacetaldoxime monooxygenase has been shown to be involved in the production of volatile organic compounds, especially phenylpropanoids and benzenoids [77]. Both enzymes have been shown to participate in pathways starting from phenylalanine, generated from Calvin cycle via shikimic acid pathway (Figure 2). Thus, if metabolic flux is toward production of anthocyanin and other secondary metabolites, pull from the phenylalanine pool may lead to a concomitant decrease in oil accumulation.

Among the 49 positional candidate genes encompassed by mPO5-1, 11 candidate genes had predicted functions related to protein and oil accumulation and/or biosynthesis (Figure 2 and Table 2). Of potential functional interest were the candidate genes annotated as transporters of either fatty acids (Glyma.05g028500) or nitrate. Fatty acids are transported across most membrane systems for modification and lipid assembly following synthesis in plastids [78]. Glyma.05g028500 may contribute to the transportation of chloroplast produced fatty acids into the peroxisome, where they can be incorporated into β-oxidation. Nitrate uptake from the soil is transcriptionally controlled by both nitrate and photosynthate availability. De Jong et al. [79] showed nitrate uptake and assimilation was closely coordinated with glucose to supply amino acids and protein for plant growth. Thus, the five candidate genes annotated as nitrate transporters may play a role in production of amino acids and total protein.

For mPO5-5, six of 27 positional candidates had predicted functions contributing to either the carbohydrate metabolic process or fatty acid activation and biosynthesis (Table 2 and Figure 2). Proteins coded by *Glyma.05g216400*, *Glyma.05g216700* and *Glyma.05g217100* are putatively involved in increasing the hexose phosphate pool, which is the backbone of the main metabolic pathways and exchange between cellular compartments [80,81,82]. The remaining three candidate genes encoded proteins for fatty acid activation and biosynthesis via triacylglycerol (TAG) degradation. Oil crops, such as soybean, oilseed rape and sunflower, store 20–60% of dry weight oil in the form of TAG [83]. These three candidate genes involved in TAG degradation may contribute to changes of oil accumulation in the seed.

Of the 23 positional candidate genes within mPO6-3, five putatively encode proteins involved in major metabolic processes, such as TAG degradation (Glyma.06g087100), gluconeogenesis (Glyma.06g087800), amino acid transportation (Glyma.06g088200 and Glyma.06g088300) and glycolysis (Glyma.06g088600) (Table 2 and Figure 2). As noted for several candidate genes in mPO5-5 (see as above), Glyma.06g087100 may control oil content in seed by TAG degradation. Glyma.06g087800 putatively encoded a malate dehydrogenase. Malate is oxidized and the resulting oxaloacetate is converted into hexoses by gluconeogenesis, so this candidate gene may contribute to increasing the hexose pool for production of amino acids, polysaccharides, other metabolic intermediates, and energy [84].

Meta-QTL mPO15-1 encompassed 49 positional candidate genes. Seven of these candidate genes putatively encode contributing factors to the biosynthesis of jasmonic acid, threonine, cell wall and alkaloid as well as degradation of protein and sucrose (Table 2 and Figure 2). Glyma.15g028200 annotated as a peroxidase is involved in active oxygen species-scavenging systems [85] and methylglyoxal degradation by Glyma.15g028900 may serve to increase the level of pyruvate [86].

Meta-QTL mPO15-2 encompassed positional 14 candidate genes, including Glyma.15g049100 and *Glyma.15g050100* (Table 2 and Figure 2). *Glyma.15g049100* putatively encodes a vinorine synthase, playing a role in alkaloid biosynthesis. *Glyma.15g050100* putatively encodes a fructose-bisphosphatase, a key enzyme in glycolysis/sucrose biosynthesis [87,88]. Thus, this gene might be involved in carbon partitioning and further regulating protein and oil content in soybean.

Among the 12 positional candidate genes encompassed by mPO15-5, notable functional candidates are *Glyma.15g142500* and *Glyma.15g143100*, which are putatively involved in cell wall degradation and acetyl-CoA biosynthesis, respectively (Table 2 and Figure 2). Enzymes in cell wall degradation of cell wall including glucan endo-1,3-β-d-glucosidase (Glyma.15g142500) involved in the complete hydrolysis of polysaccharides to glucose can lead to protein and oil accumulation [89].

Among the 26 positional candidate genes within mP20-2 and mPCM20-2 were eight functional candidates (Figure 2 and Table 2); however, only three functional candidate genes were within the further narrowed region defined by mPO20-2. Encompassed by these three meta-QTL, is *Glyma.20g024800* which putatively encodes a pyruvate kinase, the key enzyme of glycolysis. Pyruvate kinase leads to the tricarboxylic acid (TCA) cycle and provides pyruvate for the fatty acid biosynthesis in seeds [90]. In addition there are two functional candidate genes also encompassed by these three meta-QTL. Glyma.20g025200 has a putative function in ascorbic acid biosynthesis and degradation, potentially influencing the hexose phosphate pool [91], whereas Glyma.20g025400 has a putative function in asparagine biosynthesis, potentially influencing flux through the TCA cycle as well as the pool of Aspartate (Asp), a precursor to the Met and other Asp family amino acids [92].

Among the 49 positional candidates within the region of mP20-3, mO20-4, mPO20-4, and mPCM20-3 were nine putative functional candidate genes. These candidates genes putatively encode precursors to TAG biosynthesis (Glyma.20g121200, Glyma.20g121300), contributed to metabolic flux from the hexose phosphate pool (Glyma.20g120300, Glyma.20g118000, Glyma.20g122500), involved in amino acid transport (Glyma.20g121400, Glyma.20g121500), and collagen biosynthesis from its precursor, an ASP family amino acid, Lys (Glyma.20g122300, Glyma20g.122400) [92] (Table 2 and Figure 2).

## 4. Materials and Methods

### 4.1. Collection of Mapping and QTL Information for Soybean Protein, Oil, Amino Acids and Fatty Acids

Literature including scientific journals and available theses and dissertations were mined for QTL mapping studies conducted on soybean seed contents and compositions of protein and oil. Recent literature published up to and including 2016 were mined for relevant QTL mapping studies by search of Google Scholar using key words. In addition, mapping studies identified from SoyBase (available online: http://soybase.org) were also included in the study. Parents, size, crossing type and generation of population and genetic map information of each population were collected. For each QTL, name, trait, experiment location, year of experiment, names of chromosome and linkage group, LOD score, *R^2^* value (proportion of phenotypic variance explained), most likely position of QTL (in cM) and confidence interval (CI, in cM) were collected. If the CI of a QTL was not provided, the formula proposed by [93] was used for calculation of a 95% of CI,

CI = 530/(*N* × *R^2^*) (1)

CI = 163/(*N* × *R^2^*) (2)

where *N* is the size of population. CI of both backcross and F_2_ populations were calculated using equation (1), equation (2) was applied to QTL studies carried out using recombinant inbred line populations. The formula derived from [94] was used for the estimation of LOD, which is the explanatory power of a QTL, if the LOD value was not reported,

*R*^2^ = 1 − 10^(−2 LOD/N)^ (3)

All information was arranged by the name of the originating map. QTL information were discarded if *R*^2^ value is unavailable from the original study.

### 4.2. Meta-QTL Analysis

Using the soybean consensus map [44] as a reference map, QTLs for protein content, amino acid composition, oil content and fatty acid composition were projected on the reference map by BioMercator V4.2 [95]. For QTL projection on the soybean reference map, two default parameters were considered; (i) 0.25 was applied as the minimum value of the ratio of the flanking marker interval distance and (ii) 0.5 was applied as the minimum P value threshold for testing homogeneity of the flanking marker interval distances between original and consensus maps. If any QTL was not projected on the newly built map, it was excluded. 

Meta-analyses were performed to validate and refine confidence intervals of QTLs from those QTLs projected onto the reference map. This was done using BioMercator V4.2, including algorithms from MetaQTL [49]. In Meta-analysis step 1/2 [49], the projected QTLs were clustered by each chromosome using default parameters and traits for protein, oil, amino acid, fatty acid and combined, correlated traits. A total of five criteria, which are Akaike information criterion (AIC), corrected Akaike information criterion (AICc and AIC3), Bayesian information criterion (BIC) and approximate weight of evidence (AWE), were used for examination of potential meta-QTL models with the output from ClustQTL suggesting the best model for the next step. In Meta-analysis step 2/2, Meta-QTLs were generated in accordance with the best model [49].

### 4.3. Identification of Candidate Genes

Flanking/closest left or right markers of the CI of each meta-QTL were selected, only if these markers were presented on both the reference map and G. max genome assembly version Glyma.Wm82.a2.v1 (available online: http://soybase.org). Detailed information of each candidate gene was obtained from SoyBase (available online: http://soybase.org) and Phytozome v12.0 (available online: http://www.phytozome.net) for those meta-QTLs encompassing 50 or fewer positional candidate genes.

## 5. Conclusions

Comparisons of QTLs across multiple populations can be difficult due in part to the limited number of common markers shared across the populations. Our meta-analyses were able to integrate and project over 250 QTLs for seed composition onto a reference map, the soybean consensus map 4.0 [44]. Meta-analysis of QTLs helps to extricate robust loci which have been identified in multiple studies and defines an, often narrower, CI for those robust loci. In our study, the CIs for meta-QTLs were further narrowed through the incorporation of QTLs from multiple, correlated traits. In combination with a physical map (Gmax2.0), the flanking markers defining the meta-QTLs were used to identify a limited list of positional candidate genes from which functional candidates for seed composition traits could be selected based on putative functional annotations of the candidate genes. These genes provide potential targets for marker-assisted selection, fine mapping and positional cloning.

## Figures and Tables

**Table 1:** Meta-Analysis results of quantitative trait loci (QTL) clusters of trait by chromosomes.

Chr (LG) ^a^	Meta-QTL	Meta-QTL Position (cM)	CI ^b^ (cM)	Weight ^c^	Number of Projected QTL ^d^	CI of Projected QTL (cM)	Left Marker^e^				Right Marker ^e^			Physical Distance (bp)	Number of Candidate Genes ^f^
							Name	Map Position (cM)	Physical Position ^f^ (bp)		Name	Map Position (cM)	Physical Position ^f^ (bp)		
**3**	**Protein+Oil**														
**(N)**	mPO3-1	20.41	18.33–22.49	0.13	3	2.41–29.89	Satt152	17.36	3,366,405		Satt009	22.59	3,932,012	565,607	35
	mPO3-2	25.09	24.63–25.55	0.41	6	12.41–57.02	BARC-064081-18547	23.85	4,802,477		Satt530	25.97	5,664,735	862,258	56
	mPO3-3	31.05	30.22–31.88	0.21	5	12.41–57.02	BARC-065251-19285	29.93	19,028,238		BARC-011565-00290	32.46	29,807,303	10,779,065	268
	mPO3-4	58.97	58.49–59.46	0.24	3	34.68–69.21	Satt549	57.27	37,342,774		BARC-010211-00550	59.65	37,823,900	481,126	54
															
**5**	**Protein+Oil**														
**(A1)**	mPO5-1	4.84	2.43–7.25	0.08	1	2.57–7.12	BARC-040651-07808	2.45	2,295,488		BARC-019485-03631	7.66	2,748,936	453,448	49
	mPO5-2	14.61	10.52–18.71	0.08	2	10.51–27.99	BARC-044997-08863	9.48	3,924,139		Satt276	18.91	5,158,700	1,234,561	123
	mPO5-3	21.36	18.80–23.92	0.17	3	10.51–27.65	Satt276	18.91	5,158,700		BARC-014883-01912	24.07	5,758,793	600,093	51
	mPO5-4	55.73	54.18–57.28	0.50	6	47.87–62.84	BARC-037207-06739	53.77	35,375,747		BARC-040033-07641	57.47	35,961,573	585,826	65
	mPO5-5	80.34	80.20–80.49	0.17	2	76.61–82.00	Satt200	80.04	39,622,009		BARC-058653-17430	80.63	39,819,839	197,830	27
															
**6**	**Protein+Oil**														
**(C2)**	mPO6-1	5.35	3.38–7.33	0.07	1	3.37–7.33	BARC-041825-08108	2.22	1,226,605		BARC-035239-07157	11.62	1,673,727	447,122	59
	mPO6-2	31.28	29.23–33.35	0.20	3	23.02–38.62	BARC-016957-02165	26.08	3,823,755		BARC-059985-16274	34.40	5,449,370	1,625,615	209
	mPO6-3	40.84	40.28-41.40	0.27	5	23.02–43.44	BARC-027948-06704	40.25	6,712097		BARC-056271-14211	41.46	6,919,465	207,368	23
	mPO6-4	48.43	46.56–50.31	0.13	2	45.45–51.30	Satt291	42.94	7,326,519		Satt457	52.51	8,788,659	1,462,140	161
	mPO6-5	68.48	65.44–71.52	0.20	3	61.33–78.84	BARC-029937-06757	65.04	10,929,259		BARC-018663-03235	71.60	11,925,180	995,921	133
	mPO6-6	122.19	121.81–122.58	0.13	2	117.73-137.09	Sat_252	116.34	48,211,060		BARC-016969-02170	126.94	49,267,136	1,057,076	98
															
**7**	**Protein+Oil**														
**(M)**	mPO7-1	13.43	10.83–16.03	0.09	2	12.88–47.24	BARC-029703-06326	10.60	1,630781		Satt150	16.86	2,434,308	755,784	96
	mPO7-2	20.99	18.76–23.23	0.10	3	18.90–47.24	Sat_316	18.67	2,722,475		BARC-054347-12492	24.46	3,320,310	597,835	57
	mPO7-3	29.17	21.97–36.38	0.11	2	18.90–47.24	Sat_316	18.67	2,722,475		BARC-028455-05917	36.98	5,937,694	3,215,219	314
	mPO7-4	37.57	33.84–41.30	0.22	5	18.90–55.03	Satt567	32.75	4,559,651		BARC-039195-07465	41.37	6,567,400	2,007,749	199
	mPO7-5	44.44	41.84–47.04	0.39	5	18.90–55.03	BARC-042815-08424	41.37	6,443,468		BARC-048517-10647	47.38	8,461,619	2,018,151	194
	mPO7-6	66.69	57.99–75.40	0.09	1	58.99–74.40	Sat_003	57.84	12,303,557		BARC-013407-01480	75.42	31,260,765	18,957,208	590
															
**9 ^g^**	**Protein+Oil**														
**(K)**	mPO9-1	14.40	8.70–20.10	0.09	1	8.70–20.11	BARC-051589-11168	8.77	1,434,250		BARC-039923-07610	21.43	3,136,549	1,702,299	190
	mPO9-2	30.67	29.23–32.11	0.20	4	21.47–43.20	BARC-022201-04296	28.04	4,266,665		BARC-014659-01609	34.03	5,901,485	1,634,820	130
	mPO9-3	40.46	39.83–41.10	0.61	8	21.47–52.13	BARC-062013-17617	39.77	7,779,719		BARC-058145-15142	41.31	21,880,468	14,100,749	370
	mPO9-4	51.49	50.49–52.49	0.10	2	39.00–54.41	Satt725	49.08	31,346,707		Sat_044	53.24	36,759,518	5,412,811	214
															
**14 ^g^**	**Protein+Oil**														
**(B2)**	mPO14-1	17.66	14.72–20.60	0.27	3	6.09–31.73	BARC-051559-11161	14.48	2,598,423		BARC-021353-04044	21.73	4,395,242	1,796,819	201
	mPO14-2	48.70	46.81–50.60	0.62	7	38.63–61.78	BARC-064873-18956	45.46	8,132,273		BARC-055677-13598	53.92	9,110,441	978,168	74
	mPO14-3	57.12	55.44–58.80	0.11	6	38.63–61.78	BARC-014309-01312	54.51	9,434,565		Sat_182	59.99	31,281,020	21,846,455	482
															
**15 ^g^**	**Protein+Oil**														
**(E)**	mPO15-1	11.68	10.09–13.27	0.26	5	5.56–26.90	BARC-025493-06513	9.97	2,127,717		Satt411	13.66	2,517,428	389,711	49
	mPO15-2	18.98	17.96–20.01	0.40	8	5.56–31.24	BARC-008231-00112	17.95	3,964,389		BARC-042857-08439	20.04	3,846,538	(117,851)^h^	14
	mPO15-3	43.58	40.57–46.59	0.13	2	38.22–49.90	BARC-027480-06591	40.00	8,304,621		BARC-018901-03270	47.29	9,840,775	1,536,154	179
	mPO15-4	53.21	52.75–53.68	0.13	2	48.84–54.45	BARC-017283-02257	53.29	10,562,976		BARC-052667-11557	53.77	11,139,595	576,619	57
	mPO15-5	58.03	57.93–58.13	0.07	1	57.17–58.89	BARC-028607-05972	57.24	11,651,285		BARC-017755-03124	59.38	11,818,830	167,545	12
															
**19**	**Protein+Oil**														
**(L)**	mPO19-1	15.29	12.28–18.31	0.27	4	7.40–35.02	Satt446	10.42	1,678,524		Satt388	21.14	4,244,178	2,565,654	165
	mPO19-2	34.96	31.80–37.83	0.15	3	18.01–53.17	Satt497	31.39	33,865,280		BARC-013203-00448	39.34	37,425,576	3,560,296	220
	mPO19-3	50.85	48.42–53.28	0.24	4	36.20–62.21	BARC-016181-02303	46.51	38,087,635		BARC-007554-00101	54.93	39,579,279	1,491,644	121
	mPO19-4	58.94	57.31–60.58	0.17	3	36.20–62.21	BARC-059657-15973	56.99	40,154,846		Satt678	61.40	43,032,497	2,877,651	295
	mPO19-5	82.98	81.99–83.97	0.17	2	68.81–87.22	Satt664	81.33	46,227,991		BARC-014655-01607	84.05	46,596,334	368,343	54
															
**20**	**Protein**														
**(I)**	mP20-1	15.02	12.82–17.23	0.20	3	10.81–22.49	BARC-055857-13795	11.35	677,408		BARC-057033-14543	17.68	1,738,862	1,061,454	105
	mP20-2	25.43	24.37–26.50	0.60	10	10.81–39.24	Satt367	24.01	2,615,668		BARC-040841-07852	27.14	2,946,641	330,973	26
	mP20-3	49.47	49.17–49.78	0.13	3	47.33–67.38	BARC-055423-13277	44.95	36,055,353		BARC-050455-09643	49.92	36,575,544	520,191	49
	mP20-4	60.48	54.19–66.77	0.07	1	53.58–67.38	BARC-025987-05207	53.77	37,350,343		Sat_418	66.82	39,876,415	2,526,072	270
															
	**Oil**														
	mO20-1	15.86	13.56–18.16	0.19	5	11.13–34.78	BARC-055857-13795	11.35	677,408		BARC-021887-04232	18.51	1,900,702	1.223.294	111
	mO20-2	24.08	21.90–26.27	0.36	8	13.93–48.44	BARC-052017-11314	19.96	2,103,067		BARC-040841-07852	27.14	2,946,641	843,574	63
	mO20-3	35.26	29.44–41.09	0.14	3	14.28–48.44	BARC-057867-14973	29.16	28,540,212		BARC-039921-07608	42.13	35,080,674	6,540,462	307
	mO20-4	49.47	49.47–49.48	0.16	3	29.23–49.64	BARC-055423-13277	44.95	36,055,353		BARC-050455-09643	49.92	36,575,544	520,191	49
	mO20-5	64.99	62.63–67.36	0.15	2	54.48–75.56	BARC-017939-02461	60.30	38,750,487		BARC-021323-04037	67.42	39,876,415	1,125,928	115
															
	**Protein+Oil**														
	mPO20-1	15.40	13.82–16.98	0.19	9	10.81–29.58	BARC-055857-13795	11.35	677,408		BARC-057041-14548	17.03	1,666,918	989,510	100
	mPO20-2	24.98	24.07–25.90	0.46	19	10.81–48.44	Satt367	24.01	2,615,668		BARC-065829-19777	26.05	2,795,596	179,928	14
	mPO20-3	34.29	30.15–38.43	0.09	5	14.28–48.44	BARC-039387-07311	30.14	27,997,262		BARC-020713-04700	38.61	34,052,339	6,055,077	201
	mPO20-4	49.47	49.47–49.47	0.26	8	29.23–66.48	BARC-055423-13277	44.95	36,055,353		BARC-050455-09643	49.92	36,575,544	520,191	49
															
	**Protein+Cys+Met**														
	mPCM20-1	15.03	12.84–17.22	0.17	3	10.81–22.49	BARC-055857-13795	11.35	677,408		BARC-057033-14543	17.68	1,738,862	1,061,454	105
	mPCM20-2	26.09	25.28–26.90	0.56	11	10.81–39.24	Satt367	24.01	2,615,668		BARC-040841-07852	27.14	2,946,641	330,973	26
	mPCM20-3	49.47	49.40–49.54	0.17	3	47.33–67.38	BARC-055423-13277	44.95	36,055,353		BARC-050455-09643	49.92	36,575,544	520,191	49
	mPCM20-4	76.75	75.05–78.46	0.11	2	69.83–93.95	Satt292	74.78	40,623,844		Satt162	78.82	41,416,130	792,286	85
															

* Grey highlighting indicates meta-QTLs with less than 50 positional candidate genes. ^a^ Chr, chromosome; LG, linkage group ^b^ The 95% confidence interval of meta-QTL ^c^ The weights of each cluster (i.e. the mixing proportions in the mixture model) ^d^ Projected QTLs for the indicated meta-QTL ^e^ Flanking/closest left or right marker of the CI of the meta-QTL presented on both the soybean consensus map 4.0 [39] and G. max genome assembly version Glyma.Wm82.a2.v1 (Gmax2.0) (available online: http://soybase.org) ^f^ Physical positions and candidate genes are based on Gmax2.0. ^g^ Additional meta-QTLs were detected, if QTLs having a LOD score below 2.0 were also included for meta-analysis. ^h^ The orders of left and right markers were reversed on Gmax2.0.

**Table 2:** List of selected positional and functional candidate genes by meta-QTL.

Chr	Meta-QTL	Gene Name	Start (bp)	Stop (bp)	Annotation ^a^	Metabolism ^b^
3	mPO3-1		3,366,405	3,932,012		
		*Glyma.03g030400*	3,372,331	3,374,781	Phenylacetaldoxime monooxygenase	Glucosinolate biosynthesis from phenylalanine
		*Glyma.03g030500*	3,402,824	3,403,934	Chitinase	Chitinase degradation
		*Glyma.03g030600*	3,427,088	3,429,159	Phenylacetaldoxime monooxygenase	Glucosinolate biosynthesis from phenylalanine
		*Glyma.03g030800*	3,458,451	3,463,303	Phenylacetaldoxime monooxygenase	Glucosinolate biosynthesis from phenylalanine
		*Glyma.03g030900*	3,462,650	3,465,320	Phenylacetaldoxime monooxygenase	Glucosinolate biosynthesis from phenylalanine
		*Glyma.03g031000*	3,483,334	3,486,055	Phenylacetaldoxime monooxygenase	Glucosinolate biosynthesis from phenylalanine
		*Glyma.03g031200*	3,503,057	3,504,917	Phenylacetaldoxime monooxygenase	Glucosinolate biosynthesis from phenylalanine
		*Glyma.03g031300*	3,515,990	3,517,595	Costunolide synthase	Constunolide biosynthesis
		*Glyma.03g031400*	3,522,165	3,527,021	Anthocyanidin 3-*O*-glucosyltransferase	Flavonoid biosynthesis
		*Glyma.03g032500*	3,716,149	3,718,912	Anthocyanidin 3-*O*-glucosyltransferase	Flavonoid biosynthesis
		*Glyma.03g032600*	3,738,632	3,739,843	Anthocyanidin 3-*O*-glucosyltransferase	Flavonoid biosynthesis
		*Glyma.03g032700*	3,740,621	3,743,020	Anthocyanidin 3-*O*-glucosyltransferase	Flavonoid biosynthesis
		*Glyma.03g032800*	3,786,702	3,789,334	Anthocyanidin 3-*O*-glucosyltransferase	Flavonoid biosynthesis
5	mPO5-1		2,295,488	2,748,936		
		*Glyma.05g026800*	2,301,052	2,303,249	Xylogalacturonan β-1,3-xylosyltransferase	Xylogalacturonan biosynthesis
		*Glyma.05g028400*	2,444,345	2,448,119	Aldose 1-epimerase	Trehalose degradation II (trehalase)
		*Glyma.05g028500*	2,449,777	2,459,772	Lipid exporter ABCA1 and related proteins, ABC superfamily	Fatty acid transportation
		*Glyma.05g029100*	2,501,803	2,508,656	Aldehyde dehydrogenase family	Arginine degradation I (arginase pathway)/proline degradation
		*Glyma.05g029200*	2,510,682	2,517,754	Aldehyde dehydrogenase family	Arginine degradation I (arginase pathway)/proline degradation
		*Glyma.05g029900*	2,565,716	2,569,741	Nitrate transporter	Nitrate assimilation
		*Glyma.05g030300*	2,603,033	2,605,522	Nitrate transporter	Nitrate assimilation
		*Glyma.05g030400*	2,609,893	2,613,480	Nitrate transporter	Nitrate assimilation
		*Glyma.05g030500*	2,615,166	2,618,849	Nitrate transporter	Nitrate assimilation
		*Glyma.05g030600*	2,626,715	2,629,668	Nitrate transporter	Nitrate assimilation
		*Glyma.05g031200*	2,712,680	2,715,373	Glucose/Sorbosone dehydrogenase	Carbohydrate metabolic process
	mPO5-5		39,622,009	39,819,839		
		*Glyma.05g216400*	39,673,239	39,678,762	Cellulase/ENDO-1,4-BETA-GLUCANASE	Carbohydrate metabolic process
		*Glyma.05g216600*	39,686,415	39,694,722	Long-chain-fatty-acid-CoA ligase	D-*myo*-inositol (1,4,5)-trisphosphate biosynthesis/fatty acid activation
		*Glyma.05g216700*	39,696,647	39,699,812	Nucleoside-diphosphate kinase	UTP and CTP *de novo* biosynthesis
		*Glyma.05g217100*	39,735,139	39,739,763	UDP-glucose 4-epimerase	Galactose degradation I (Leloir pathway)
		*Glyma.05g217400*	39,752,430	39,754,981	Lipase (class 3)	Fatty acid biosynthesis
		*Glyma.05g217600*	39,762,157	39,765,360	1-phosphatidylinositol 4-kinase	D-*myo*-inositol (1,4,5)-trisphosphate biosynthesis/3-phosphoinositide biosynthesis
6	mPO6-3		6,712,097	6,919,465		
		*Glyma.06g087100*	6,750974	6,754,953	Acylglycerol lipase	Triacylglycerol degradation
		*Glyma.06g087800*	6,788436	6,793,747	Malate dehydrogenase (oxaloacetate-decarboxylating) (NADP^+^)	Gluconeogenesis
		*Glyma.06g088200*	6,824021	6,828,704	Amino acid permease 1	Amino acid transportation
		*Glyma.06g088300*	6,838983	6,846,287	Amino acid permease 8	Amino acid transportation
		*Glyma.06g088600*	6,865939	6,873,001	6-phosphofructokinase	Glycolysis
15	mPO15-1 ^c^		2,127,717	2,517,428		
		*Glyma.15g026400*	2,130531	2,134,563	Linoleate 9*S-*lipoxygenase	Jasmonic acid biosynthesis
		*Glyma.15g026500*	2,142191	2,147,489	Linoleate 9*S-*lipoxygenase	Jasmonic acid biosynthesis
		*Glyma.15g027000*	2,171688	2,174,398	Threonine synthase	Threonine biosynthesis
		*Glyma.15g027100*	2,177956	2,179,285	Glucuronosyl-*N-*acetylglucosaminyl-proteoglycan 4-α-*N-*acetylglucosaminyltransferase	Cell wall synthesis
		*Glyma.15g028200*	2,254166	2,256,553	Peroxidase	Active oxygen species-scavenging systems
		*Glyma.15g028900*	2,325622	2,329,211	Hydroxyacylglutathione hydrolase	Methylglyoxal degradation
		*Glyma.15g029200*	2,356317	2,366,181	Electron-transferring-flavoprotein dehydrogenase	Protein degradation
		*Glyma.15g029300*	2,366688	2,369,189	Strictosidine synthase	Alkaloid biosynthesis
		*Glyma.15g029800*	2,387957	2,391,720	Fructokinase	Sucrose degradation
	mPO15-2 ^c^		3,846,538	3,964,389		
		*Glyma.15g049100*	3,869,436	3,870,986	Vinorine synthase	Alkaloid biosynthesis
		*Glyma.15g050100*	3,958,743	3,960,926	Fructose-bisphosphatase	Glycolysis/Sucrose biosynthesis
	mPO15-5 ^c^		11,651,285	11,818,830		
		*Glyma.15g142500*	11,655,353	11,656,913	Glucan endo-1,3-β-d-glucosidase	Cell wall degradation
		*Glyma.15g143100*	11,744,760	11,750,567	Dihydrolipoyl dehydrogenase	Acetyl-CoA biosynthesis
20	mP20-2,		2,615,668	2,946,641		
	mPCM20-2	*Glyma.20g024800*	2,679,571	2,683,022	Pyruvate kinase	Glycolysis
		*Glyma.20g025200*	2,743,092	2,745,282	l-ascorbate oxidase	Ascorbic acid biosynthesis/degradation
		*Glyma.20g025400*	2,768,770	2,781,380	Asparagine synthase (glutamine-hydrolyzing)	Asparagine biosynthesis
		*Glyma.20g025700*	2,813,089	2,825,575	2-hydroxy-6-oxonona-2,4-dienedioate hydrolase	TCA cycle
		*Glyma.20g026000*	2,856,949	2,863,351	Sarcosine/dimethylglycine *N-*methyltransferase	Amino acid biosynthesis
		*Glyma.20g026300*	2,894,580	2,905,241	β-glucosidase	Starch degradation
		*Glyma.20g026400*	2,909,731	2,912,529	UDP-glucose 6-dehydrogenase	l-ascorbate biosynthesis
		*Glyma.20g026700*	2,935,653	2,945,665	Phosphorylase	Carbohydrate metabolism
	mPO20-2		2,615,668	2,795,596		
		*Glyma.20g024800*	2,679,571	2,683,022	Pyruvate kinase	Glycolysis
		*Glyma.20g025200*	2,743,092	2,745,282	l-ascorbate oxidase	Ascorbic acid biosynthesis/degradation
		*Glyma.20g025400*	2,768,770	2,781,380	Asparagine synthase (glutamine-hydrolyzing)	Asparagine biosynthesis
	mP20-3,		36,055,353	36,575,544		
	mO20-4,	*Glyma.20g118000*	36,083,279	36,091,841	β-lactamase	Cell wall metabolism
	mPO20-4,	*Glyma.20g120300*	36,306,759	36,311,703	Trehalose-phosphatase	Trehalose biosynthesis
	mPCM20-3	*Glyma.20g121200*	36,384,081	36,391,030	Diacylglycerol diphosphate phosphatase	Triacylglycerol biosynthesis
		*Glyma.20g121300*	36,395,415	36,400,718	Diacylglycerol diphosphate phosphatase	Triacylglycerol biosynthesis
		*Glyma.20g121400*	36,402,262	36,406,832	Amino acid permease	Amino acid transportation
		*Glyma.20g121500*	36,414,549	36,419,060	Amino acid permease	Amino acid transportation
		*Glyma.20g122300*	36,506,583	36,511,326	Procollagen-lysine 5-dioxygenase	Collagen biosynthesis via Lysine
		*Glyma.20g122400*	36,516,501	36,521,436	Procollagen-lysine 5-dioxygenase	Collagen biosynthesis via Lysine
		*Glyma.20g122500*	36,524,182	36,526,957	Fructose-bisphosphate aldolase	Sucrose biosynthesis

^a^ Annotation by SoyCyc7.0 (available online: http://www.plantcyc.org) or PTHR (available online: http://www.patherdb.org); ^b^ Metabolism involved based on annotation; ^c^ Additional meta-QTLs were detected, if QTLs having a LOD score below 2.0 were also included for meta-analysis.

## Supplementary Materials

Supplementary materials can be found at www.mdpi.com/1422-0067/18/6/1180/s1.

## Abbreviations

AIC	Akaike information criterion
AICc	Corrected Akaike information criterion
AIC_3_	Corrected Akaike information criterion
Asp	Aspartate
AWE	Approximate weight of evidence
BIC	Bayesian information criterion
Chr	Chromosome
CI	Confidence interval
cM	Centimorgan
Cys	Cysteine
FAD2	Microsomal oleate desaturase
FAD3	ω-3 fatty-acid desaturase
Gmax2.0	Glyma.Wm82.a2.v1
KAS II	3-keto-acyl-ACP synthase II
kb	Kilobase
LOD	Logarithm (base 10) of odds
LG	Linkage group
Lys	Lysine
Met	Methionine
mO	Meta-QTL for oil
mP	Meta-QTL for protein
mPO	Meta-QTL for protein and oil in combination
mPCM	Meta-QTL for protein, cysteine, and methionine in combination
PTHR	Protein analysis through evolutionary relationships
QTL	Quantitative trait loci
SACPD-C	Δ^9^-stearoyl-acyl-carrier-protein-desaturase
SoyBase	SoyBase and the Soybean Breeder’s Toolbox
SoyCys7.0	Soybean metabolic pathway reference database
TAG	Triacylglycerol
TCA	Tricarboxylic acid
Thr	Threonine
