# Supplementary material for: Meta-Analyses of QTLs Associated with Protein and Oil Contents and Compositions in Soybean [Glycine max (L.) Merr.] Seed
Source: Int J Mol Sci. 2017 Jun 1;18(6):1180. doi: 10.3390/ijms18061180 (PMC5486003; doi:10.3390/ijms18061180)

Article

Meta-analysis of QTL Associated with Protein and Oil Contents and Compositions in Soybean [Glycine max (L.) Merr.] Seed

Kyujung Van and Leah K. McHale *

**Table S1.** List of references used for this study.

**Showed in the suppletable excel.**

**Table S2.** Number of collected QTLs and QTLs used for this study by trait.

| **Trait** |  | **No. of Collected QTLs** | **No. of QTLs Used for Meta-Analysis** | |
| --- | --- | --- | --- | --- |
|  |  |  | All QTLs included | LOD > 2.0 |
| Protein |  | 355 | 184 | 175 |
| Oil |  | 367 | 212 | 205 |
| Amino acids |  | 358 | 156 | 156 |
|  | Ala | 15 | 10 | 10 |
|  | Arg | 12 | 5 | 5 |
|  | Asp | 14 | 9 | 9 |
|  | Cys | 34 | 12 | 12 |
|  | Gln | 7 | 0 | 0 |
|  | Glu | 8 | 8 | 8 |
|  | Gly | 12 | 7 | 7 |
|  | His | 9 | 3 | 3 |
|  | Ile | 14 | 8 | 8 |
|  | Leu | 18 | 9 | 9 |
|  | Lys | 26 | 5 | 5 |
|  | Met | 38 | 12 | 12 |
|  | Phe | 17 | 9 | 9 |
|  | Pro | 15 | 9 | 9 |
|  | Ser | 15 | 11 | 11 |
|  | Thr | 39 | 13 | 13 |
|  | Trp | 31 | 9 | 9 |
|  | Tyr | 17 | 9 | 9 |
|  | Val | 17 | 8 | 8 |
| Fatty acids |  | 240 | 120 | 113 |
|  | Linoleic acid | 45 | 21 | 19 |
|  | Linolenic acid | 74 | 39 | 37 |
|  | Oleic acid | 46 | 21 | 21 |
|  | Palmitic acid | 45 | 22 | 19 |
|  | Stearic acid | 30 | 17 | 17 |
| Total |  | 1,320 | 672 | 649 |

**Table S3.** The best meta-QTL models by chromosomes and traits.

| Chr | Linkage Group | No. of Projected QTLs | | The Best Meta-QTL Models^a^ | | | | | | |
| --- | --- | --- | --- | --- | --- | --- | --- | --- | --- | --- |
|  |  | **All QTLs** | **LOD > 2.0** | **Protein** | **Oil** | **AA** | **FA** | **Protein+Oil** | **Protein**  **+Cys+Met** | **Misc. Information** |
|  |  |  | |  |  |  |  |  |  |  |
| 1 | D1a | 4 | 4 | -^b^ | 1 | - | - | 1 | - | No projected protein QTL,  1 projected AA QTL (Thr) |
| 2 | D1b | 14 | 12 | 4 | - | - | 3 | 3 | - | No projected AA QTL |
| 3 | N | 14 | 14 | 3 | 3 | - | 2 | 4^c^ | - | No projected AA QTL |
| 4 | C1 | 8 | 8 | 5 | 2 | - | - | 4 | - | No projected AA & FA QTL |
| 5 | A1 | 20 | 20 | 4 | 4 | - | 3 | 5^c^ | - | No projected AA QTL |
| 6 | C2 | 26 | 26 | 4 | 5 | - | 2 | 6^c^ | 4 | Only 1 projected QTL (Met) |
| 7 | M | 12 | 12 | 3 | 5 | - | - | 6^c^ | - | 1 FA QTL, No projected AA QTL |
| 8 | A2 | 5 | 5 | 1 | 2 | - | - | 2 | - | 1 projected AA QTL (Lys) |
| 9 | K | 17 | 14 | 2 | 3 | 1 | 3 | 3^d^ | 3 | 2 projected AA QTL (Met & Thr) |
| 10 | O | 12 | 12 | 2 | 4 | - | 3 | 3 | - | No projected AA QTL |
| 11 | B1 | 3 | 3 | 2 | - | - | - | 3 | - | 2 projected protein QTLs,  1 projected oil QTL only |
| 12 | H | 6 | 6 | - | 3 | - | 1 | 2 | - | 1 protein QTL,  No projected AA QTL |
| 13 | F | 15 | 14 | 4 | 3 | - | 3 | 4 | - | No projected AA QTL |
| 14 | B2 | 16 | 14 | 3 | 2 | - | 2 | 3^d^ | - | No projected AA QTL |
| 15 | E | 21 | 16 | 3 | 3 | - | 3 | 5^d^ | - | No projected AA QTL |
| 16 | J | 5 | 4 | - | - | - | 2 | - | - | Projected FA QTLs only |
| 17 | D2 | 13 | 11 | 2 | 3 | - | 3 | 3 | - | No projected AA QTL |
| 18 | G | 18 | 17 | 4 | 3 | - | 5 | 5 | - | No projected AA QTL |
| 19 | L | 17 | 16 | 4 | 5 | - | 2 | 5^c^ | - | No projected AA QTL |
| 20 | I | 38 | 35 | 4^c^ | 5^c^ | 5 | 1 | 4^c^ | 4^c^ | 5 projected AA QTLs |
| Total | | 284 | 263 |  |  |  |  |  |  |  |
|  | |  | |  |  |  |  |  |  |  |

^a^ BioMercator v4.2 evaluated all meta-QTL models (1 to *n*), where *n* is the total number of QTLs identified in each QTL cluster and suggested the best model by each chromosome and traits. ^b^ No model was suggested by meta-QTL (Meta-analysis 1 of 2, [44]) because no or few QTLs corresponding to the trait were projected on the chromosome. ^c^ The most likely meta-QTL model given position and confidence interval by meta-QTL (Meta-analysis 2 of 2, [44]), if all collected QTLs or QTLs with LOD scores > 2.0 were used for meta-analysis. ^d^ The most likely meta-QTL model given position and confidence interval by meta-QTL, if only QTLs with LOD scores > 2.0 were used for meta-analysis.

**Table S4.** The most appropriate meta-QTL models for various traits suggested by five criteria.

| Chr^a^ | Linkage group | The best model (K) ^b^ and values by model selection criteria | | | | | | | | | |
| --- | --- | --- | --- | --- | --- | --- | --- | --- | --- | --- | --- |
|  |  | AIC^c^ | | AICc^d^ | | AIC3^e^ | | BIC^f^ | | AWE^g^ | |
|  |  | K | Value | K | Value | K | Value | K | Value | K | Value |
|  |  |  |  |  |  |  |  |  |  |  |  |
| Protein only | | | | | | | | | | | |
| 20 | I | 4 | 93.79 | 3 | 102.46 | 4 | 100.79 | 4 | 98.75 | 3 | 99.96 |
|  |  |  |  |  |  |  |  |  |  |  |  |
| Oil only | | | | | | | | | | | |
| 20 | I | 5 | 103.37 | 4 | 126.35 | 4 | 110.95 | 4 | 107.91 | 4 | 117.46 |
|  |  |  |  |  |  |  |  |  |  |  |  |
| Protein+Oil | | | | | | | | | | | |
| 3 | N | 4 | 80.53 | 3 | 102.50 | 4 | 87.53 | 4 | 83.31 | 4 | 96.31 |
| 5 | A1 | 5 | 93.08 | 4 | 121.24 | 4 | 100.24 | 4 | 96.63 | 4 | 106.26 |
| 6 | C2 | 6 | 123.67 | 5 | 205.97 | 6 | 134.67 | 6 | 131.46 | 6 | 143.29 |
| 7 | M | 6 | 89.50 | 4 | 149.72 | 5 | 98.90 | 5 | 93.48 | 5 | 113.72 |
| 9^h^ | K | 4 | 78.92 | 3 | 112.22 | 4 | 85.92 | 4 | 81.70 | 4 | 99.17 |
| 14^h^ | B2 | 3 | 76.70 | 2 | 87.41 | 3 | 81.70 | 3 | 78.69 | 3 | 87.59 |
| 15^h^ | E | 5 | 108.41 | 5 | 144.41 | 5 | 117.41 | 5 | 114.79 | 5 | 123.98 |
| 19 | L | 5 | 110.71 | 4 | 156.39 | 5 | 119.71 | 5 | 115.07 | 5 | 128.65 |
| 20 | I | 4 | 229.11 | 3 | 234.41 | 4 | 236.11 | 3 | 238.34 | 3 | 217.74 |
|  |  |  |  |  |  |  |  |  |  |  |  |
| Protein+Cys+Met | | | | | | | | | | | |
| 20 | I | 4 | 134.03 | 4 | 145.23 | 4 | 141.03 | 4 | 140.27 | 4 | 139.88 |
|  |  |  |  |  |  |  |  |  |  |  |  |

^a^ Chromosomes (Chrs) having successfully positioned meta-QTLs were only shown in this table. ^b^ BioMercator v4.2 evaluated all meta-QTL models (1 to *n*), where *n* is the total number of QTLs identified in each QTL cluster and suggested the best model by each chromosome. ^c^ AIC: Akaike information criterion. ^d^ AICc and AIC3: corrected Akaike information criterion. ^e^ BIC: Bayesian information criterion. ^f^ AWE: Approximate weight of evidence. **^g^** The most likely meta-QTL model given position and confidence interval by meta-QTL, if only QTLs with LOD > 2.0 were used for meta-analysis.

**Table S5.** Detailed information of candidate genes from each meta-QTL (< 50 candidate genes) obtained from SoyBase (available online: http://soybase.org) and Phytozome v12.0 (available online: <http://www.phytozome.net>).

Showed in the suppletable excel.


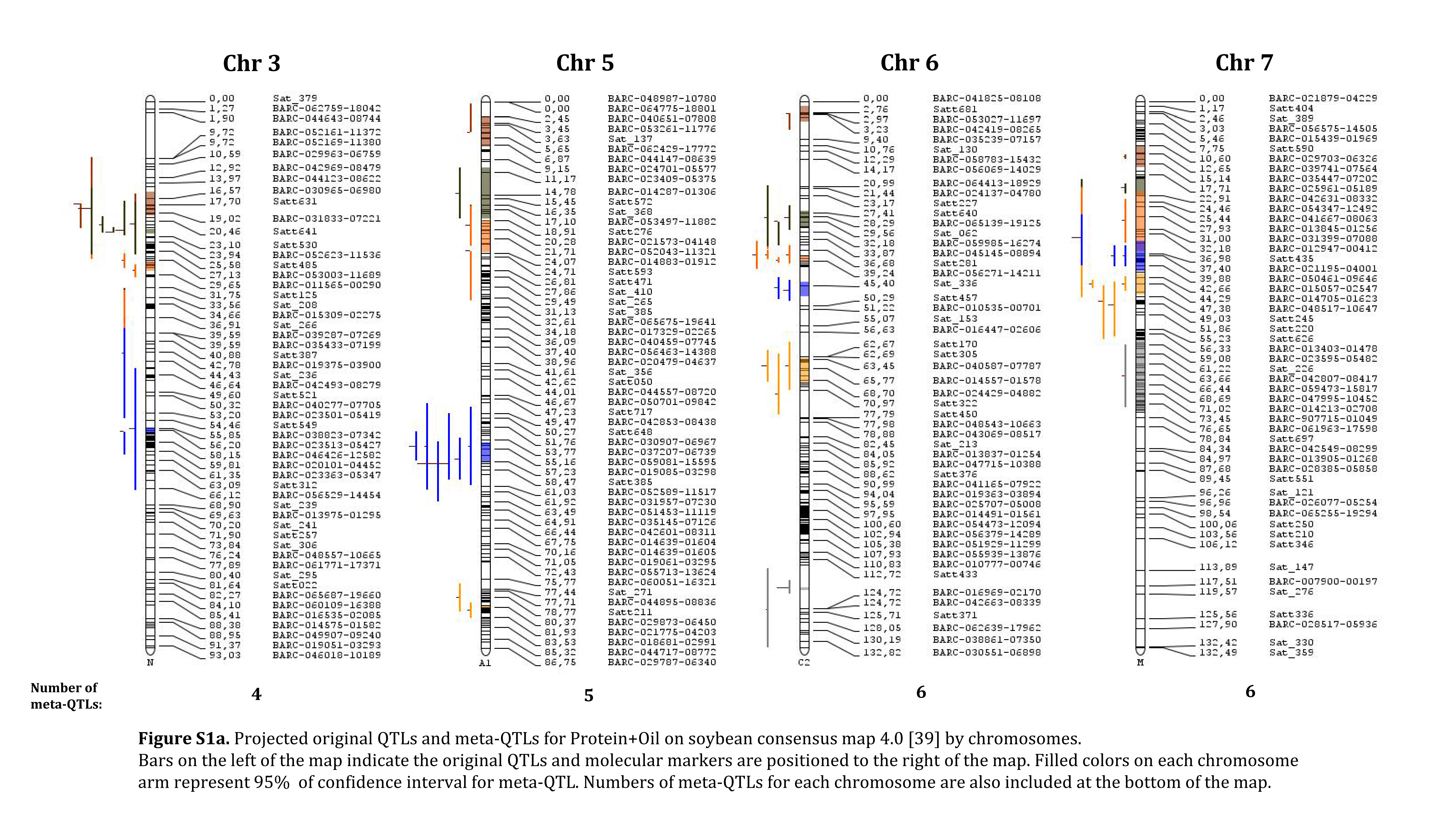


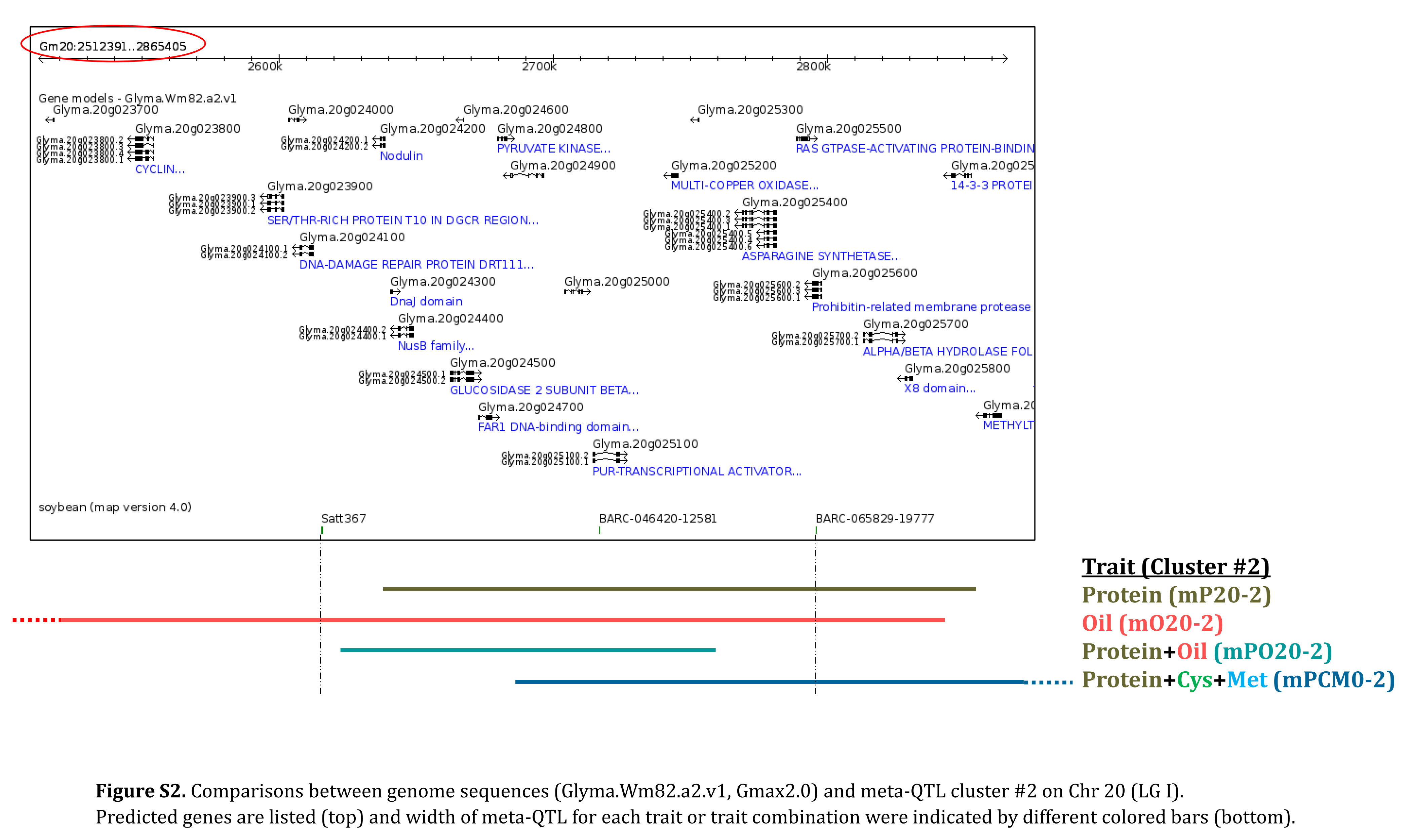

Supplement: Supplementary file 1 [file ijms-18-01180-s001.zip › ijms-196880-suppletables/1-ijms-196880-suppletables .docx]
