# Supplementary material for: Meta-Analyses of QTLs Associated with Protein and Oil Contents and Compositions in Soybean [Glycine max (L.) Merr.] Seed
Source: Int J Mol Sci. 2017 Jun 1;18(6):1180. doi: 10.3390/ijms18061180 (PMC5486003; doi:10.3390/ijms18061180)
Supplement: Supplementary file 1 [file ijms-18-01180-s001.zip › ijms-196880-suppletables/ijms-196880-supplementary.pdf]

Article

Meta-analysis of QTL Associated with Protein and Oil Contents and Compositions in Soybean [Glycine max (L.) Merr.] Seed

Kyujung Van and Leah K. McHale *

Table S1. List of references used for this study.

Showed in the suppletable excel.

Table S2. Number of collected QTLs and QTLs used for this study by trait.

Trait		No. of Collected QTLs	No. of QTLs Used for Meta-Analysis	
			All QTLs included	LOD > 2.0
Protein		355	184	175
Oil		367	212	205
Amino acids		358	156	156
	Ala	15	10	10
	Arg	12	5	5
	Asp	14	9	9
	Cys	34	12	12
	Gln	7	0	0
	Glu	8	8	8
	Gly	12	7	7
	His	9	3	3
	Ile	14	8	8
	Leu	18	9	9
	Lys	26	5	5
	Met	38	12	12
	Phe	17	9	9
	Pro	15	9	9
	Ser	15	11	11
	Thr	39	13	13
	Trp	31	9	9
	Tyr	17	9	9
	Val	17	8	8
Fatty acids		240	120	113
	Linoleic acid	45	21	19
	Linolenic acid	74	39	37
	Oleic acid	46	21	21
	Palmitic acid	45	22	19
	Stearic acid	30	17	17
Total		1,320	672	649

Table S3. The best meta-QTL models by chromosomes and traits.

Chr	Linkage Group	No. of Projected QTLs		The Best Meta-QTL Models[a]						
		All QTLs	LOD > 2.0	Protein	Oil	AA	FA	Protein+Oil	Protein +Cys+Met	Misc. Information
1	D1a	4	4	-[b]	1	-	-	1	-	No projected protein QTL, 1 projected AA QTL (Thr)
2	D1b	14	12	4	-	-	3	3	-	No projected AA QTL
3	N	14	14	3	3	-	2	4[c]	-	No projected AA QTL
4	C1	8	8	5	2	-	-	4	-	No projected AA & FA QTL
5	A1	20	20	4	4	-	3	5[c]	-	No projected AA QTL
6	C2	26	26	4	5	-	2	6[c]	4	Only 1 projected QTL (Met)
7	M	12	12	3	5	-	-	6[c]	-	1 FA QTL, No projected AA QTL
8	A2	5	5	1	2	-	-	2	-	1 projected AA QTL (Lys)
9	K	17	14	2	3	1	3	3[d]	3	2 projected AA QTL (Met & Thr)
10	O	12	12	2	4	-	3	3	-	No projected AA QTL
11	B1	3	3	2	-	-	-	3	-	2 projected protein QTLs, 1 projected oil QTL only
12	H	6	6	-	3	-	1	2	-	1 protein QTL, No projected AA QTL
13	F	15	14	4	3	-	3	4	-	No projected AA QTL
14	B2	16	14	3	2	-	2	3[d]	-	No projected AA QTL
15	E	21	16	3	3	-	3	5[d]	-	No projected AA QTL
16	J	5	4	-	-	-	2	-	-	Projected FA QTLs only
17	D2	13	11	2	3	-	3	3	-	No projected AA QTL
18	G	18	17	4	3	-	5	5	-	No projected AA QTL
19	L	17	16	4	5	-	2	5[c]	-	No projected AA QTL
20	I	38	35	4[c]	5[c]	5	1	4[c]	4[c]	5 projected AA QTLs
**Total**		284	263							

[a] BioMercator v4.2 evaluated all meta-QTL models (1 to *n*), where *n* is the total number of QTLs identified in each QTL cluster and suggested the best model by each chromosome and traits. [b] No model was suggested by meta-QTL (Meta-analysis 1 of 2, [44]) because no or few QTLs corresponding to the trait were projected on the chromosome. [c] The most likely meta-QTL model given position and confidence interval by meta-QTL (Meta-analysis 2 of 2, [44]), if all collected QTLs or QTLs with LOD scores > 2.0 were used for meta-analysis. [d] The most likely meta-QTL model given position and confidence interval by meta-QTL, if only QTLs with LOD scores > 2.0 were used for meta-analysis.

Table S4. The most appropriate meta-QTL models for various traits suggested by five criteria.

Chr[a]	Linkage group	The best model (K) [b] and values by model selection criteria									
		AIC[c]		AICc[d]		AIC3[e]		BIC[f]		AWE[g]	
		K	Value	K	Value	K	Value	K	Value	K	Value
**Protein only**											
20	I	4	93.79	3	102.46	4	100.79	4	98.75	3	99.96
**Oil only**											
20	I	5	103.37	4	126.35	4	110.95	4	107.91	4	117.46
**Protein+Oil**											
3	N	4	80.53	3	102.50	4	87.53	4	83.31	4	96.31
5	A1	5	93.08	4	121.24	4	100.24	4	96.63	4	106.26
6	C2	6	123.67	5	205.97	6	134.67	6	131.46	6	143.29
7	M	6	89.50	4	149.72	5	98.90	5	93.48	5	113.72
9[h]	K	4	78.92	3	112.22	4	85.92	4	81.70	4	99.17
14[h]	B2	3	76.70	2	87.41	3	81.70	3	78.69	3	87.59
15[h]	E	5	108.41	5	144.41	5	117.41	5	114.79	5	123.98
19	L	5	110.71	4	156.39	5	119.71	5	115.07	5	128.65
20	I	4	229.11	3	234.41	4	236.11	3	238.34	3	217.74
**Protein+Cys+Met**											
20	I	4	134.03	4	145.23	4	141.03	4	140.27	4	139.88

[a] Chromosomes (Chrs) having successfully positioned meta-QTLs were only shown in this table. [b] BioMercator v4.2 evaluated all meta-QTL models (1 to n), where n is the total number of QTLs identified in each QTL cluster and suggested the best model by each chromosome. [c] AIC: Akaike information criterion. [d] AICc and AIC3: corrected Akaike information criterion. [e] BIC: Bayesian information criterion. [f] AWE: Approximate weight of evidence. [g] The most likely meta-QTL model given position and confidence interval by meta-QTL, if only QTLs with LOD > 2.0 were used for meta-analysis.

Table S5. Detailed information of candidate genes from each meta-QTL (< 50 candidate genes) obtained from SoyBase (available online: http://soybase.org) and Phytozome v12.0 (available online: http://www.phytozome.net).

Showed in the suppletable excel.
